# Infants’ Somatotopic Neural Responses to Seeing Human Actions: I’ve Got You under My Skin

**DOI:** 10.1371/journal.pone.0077905

**Published:** 2013-10-30

**Authors:** Joni N. Saby, Andrew N. Meltzoff, Peter J. Marshall

**Affiliations:** 1 Department of Psychology, Temple University, Philadelphia, Pennsylvania, United States of America; 2 Institute for Learning & Brain Sciences, University of Washington, Seattle, Washington, United States of America; University of Rome, Italy

## Abstract

Human infants rapidly learn new skills and customs via imitation, but the neural linkages between action perception and production are not well understood. Neuroscience studies in adults suggest that a key component of imitation–identifying the corresponding body part used in the acts of self and other–has an organized neural signature. In adults, perceiving someone using a specific body part (e.g., hand vs. foot) is associated with activation of the corresponding area of the sensory and/or motor strip in the observer’s brain–a phenomenon called *neural somatotopy*. Here we examine whether preverbal infants also exhibit somatotopic neural responses during the observation of others’ actions. 14-month-old infants were randomly assigned to watch an adult reach towards and touch an object using either her hand or her foot. The scalp electroencephalogram (EEG) was recorded and event-related changes in the sensorimotor mu rhythm were analyzed. Mu rhythm desynchronization was greater over hand areas of sensorimotor cortex during observation of hand actions and was greater over the foot area for observation of foot actions. This provides the first evidence that infants’ observation of someone else using a particular body part activates the corresponding areas of sensorimotor cortex. We hypothesize that this somatotopic organization in the developing brain supports imitation and cultural learning. The findings connect developmental cognitive neuroscience, adult neuroscience, action representation, and behavioral imitation.

## Introduction

There is burgeoning interest in the interface connecting neuroscience and social cognition [1]–[3]. A foundational topic in social-cognitive neuroscience concerns linkages between the neural systems involved in processing others’ actions and those involved in producing and monitoring one’s own actions. One impetus for studies in this area is the finding of neurons in the premotor cortex (F5) of macaque monkeys that are active during both the observation and execution of actions [4], [5]. More recently, various neuroimaging methods have been used to investigate related issues in humans [6].

In humans, functional magnetic resonance imaging (fMRI) studies of adults have shown a somatotopic response to action observation in premotor and somatosensory cortex [7]–[9]. This work supports the idea that the actions of others are processed in relation to one’s own bodily action systems, although the nature and extent of such influences are under active debate [10]. Developmental data can play a valuable role in informing this debate, but empirical neuroscience studies of infant populations remain sparse. Psychologists have amassed a great deal of data concerning goal-directed behavior in human infancy, which has led to interest in aligning behavioral work with developmental neuroscientific studies [11], [12].

The domain of infant action processing provides an opportunity for linking developmental neuroscience to behavioral work on infant imitation and social-cognitive learning [13]. Infants’ imitation of others’ actions is influenced by a variety of factors, including the specific means by which an observed action is carried out. For example, 14-month-old infants can imitate the novel act of using their heads to push an object to activate it [14], suggesting that the specific effector used to accomplish a goal is preserved in infants’ action representations. A similar mapping between the corresponding body parts of self and other–*organ identification*–is a crucial part of cognitive models of infant imitation [15].

Little is known about how infant brain responses during action observation might vary as a function of the specific effector the actor uses to accomplish a goal. Do infants exhibit neural somatotopy? Is the observation of actions performed by another person’s body part associated with activation of the corresponding area of the sensory and/or motor strip? A somatotopic pattern of cortical activation has been reported for infants’ execution of limb movements and in response to direct tactile stimulation of the infant’s body [16], but no prior infant study has investigated the possibility of somatotopy during *action observation* alone. Such an organization of the developing brain could facilitate infants’ mapping between the acts of self and other–a mapping that is a fundamental component of imitation, interpersonal identification, and cultural learning [15], [17].

Developmental cognitive neuroscientists have utilized electroencephalographic (EEG) methods to study how observed actions are processed in the infant brain [18], with a focus on changes in band power during infants’ observation and execution of action. The infant EEG can be decomposed into functionally distinct frequency bands [19], [20] and recent studies have focused on the mu rhythm at central electrode sites as an index of activation of underlying sensorimotor cortex [21]. The mu rhythm oscillates in the alpha frequency range but is distinct from the visual alpha rhythm at posterior sites [22]. The infant mu rhythm is desynchronized (reduced in amplitude) both during the execution of actions and the observation of actions performed by others [23]–[25], with desynchronization being greater for the observation of goal-directed compared to mimed actions [26], [27].

Here we examine whether the response of the infant mu rhythm shows a somatotopic pattern during observation of another person’s action. In adult studies, the mu rhythm shows a somatotopic response such that imagined and executed hand movements are associated with greater mu desynchronization at central electrodes overlying hand regions of sensorimotor cortex (C3 and C4) than over the foot area (Cz). Conversely, for foot actions mu desynchronization is greater over the foot area than over hand areas [28]–[31]. The adult mu rhythm is also desynchronized over the hand area for anticipated tactile stimulation of the hand, but not for anticipated tactile stimulation of the foot [32].

Two groups of infants observed an actor accomplish the same goal using either her hand or her foot. We predicted that infants observing hand actions would exhibit greater desynchronization at electrodes overlying hand areas of sensorimotor cortex (C3, C4) than at the electrode overlying the foot area (Cz). For infants observing foot actions, the opposite pattern was predicted.

## Methods

### Ethics Statement

All study procedures were approved by the Institutional Review Board at Temple University. Written informed consent was provided by the infant’s parent or guardian prior to the start of the experiment.

### Participants

Seventy 14-month-old infants and their families were recruited from a diverse urban environment using mailing lists. Prior to scheduling families for participation in the study, parents were asked about their infant’s health and development, including specific questions about medical problems noted at birth, developmental delays, and medication use. To participate, infants had to be born at term, have parents who were not both left-handed, and be free of chronic developmental problems. Prior to participation, infants were randomly assigned to one of two independent groups: Observe-Hand or Observe-Foot. The EEG analyses were carried out for 32 infants (*M* age = 62.4 weeks, *SD* = 1.4; 19 male, *n* = 15 for Observe-Hand and *n* = 17 for Observe-Foot). The remaining infants were excluded due to technical error (*n* = 5) or an insufficient number of trials (<8) that were free of artifact and during which the infant was still and attentive (*n = *33). This rate of data loss is similar to other studies of infants’ action processing involving the EEG mu rhythm [33]–[35].

### Procedure

**Figure 1:**
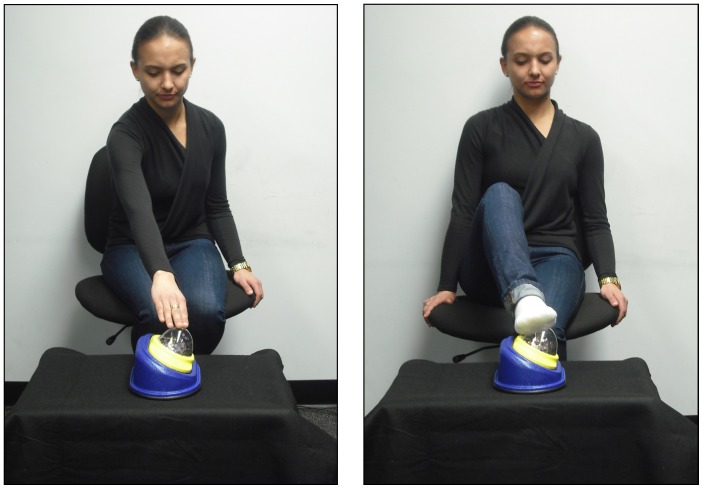
Experimental stimuli. These photographs show the experimental setup for the Observe-Hand and Observe-Foot groups. The actor in the photograph has given written informed consent, as outlined in the PLOS consent form, to publication of their photograph.

All visits to the laboratory took place in the morning. Participants were fitted with an EEG cap and were seated on their caregiver’s lap facing an experimenter who was seated 2 m away. Trials involved watching the experimenter act on a particular toy which has a clear plastic dome mounted on a sturdy base (Sensory Dome, Achievement Products; see Figure 1). This specific object is unfamiliar yet engaging to infants, and can be activated with a single hand or foot. When light pressure was applied to the top of the object, a musical sequence played and a spinning rotor disturbed multi-colored confetti until the pressure was released. The object was located on a table in front of the experimenter, in the view of the infant. The height of the table (53 cm) was such that the object occupied an intermediate position between the experimenter’s hand and foot, allowing a naturalistic reach using either effector. This experimental procedure was designed to hold constant the goal and the resulting effect (pressing the button to activate the toy) while systematically varying the body part the infant saw being used to accomplish this goal.

The experimental protocol comprised blocks of four observation trials during which the experimenter reached for and pressed the dome object either using her right hand (Observe-Hand group) or her right foot (Observe-Foot group). The object was activated for 2 s in each trial, after which the experimenter brought her hand or foot back to a resting position. The mean interval between the onset of the object activation in one trial and the same time point in the next trial was 5.5 s. Blocks of observation trials were presented until the infant was no longer attending to the experimenter’s demonstrations. The blocks were separated by a 30 s period in which infants viewed clips from a commercially available animation video as alternative stimulation from the repetitive observation trials.

The experimental session was videotaped, with a vertical interval time code (VITC) placed on the video signal that was aligned with the EEG collection to the precision of one NTSC video frame (33 ms). Laboratory control software (James Long Company, Caroga Lake, NY) was used to simultaneously trigger the onset of the EEG collection and the onset of VITC generation. Videos were coded offline and specific frames were marked corresponding to the onset of the experimenter’s reach towards the object, and the point at which the experimenter’s hand or foot made contact with the object. Videos were also coded for infant attention and motor movement. Trials in which infants did not attend to the experimenter’s action or in which infants moved their trunk, hands or feet were excluded from the EEG analysis. The laboratory protocol included calibration stimuli that were visible on the video record and which also generated a response in the EEG data stream. The comparison of timing from these stimuli enabled precise adjustment of the sampling rate used in the offline EEG analyses, ensuring consistent temporal alignment between the EEG and video signals.

### EEG Collection and Processing

EEG was recorded from 28 scalp sites using a lycra stretch cap designed for use with infants (Electro-Cap International, Eaton, OH). Electrode impedances were accepted if they were below 35 kΩ. The EEG signals were amplified using optically isolated, high input impedance (>1 GΩ) custom bioamplifiers (SA Instrumentation, San Diego, CA) and were digitized at 512 Hz using a 16-bit A/D converter (+/−5 V input range). Bioamplifier gain was 4000 and the hardware filter (12 dB/octave rolloff) settings were.1 Hz (high-pass) and 100 Hz (low-pass). The signal was collected referenced to the vertex (Cz) with an AFz ground and was subsequently re-referenced to an average mastoids configuration. Data processing and analysis was carried out using the EEGLAB toolbox for MATLAB [36] in combination with software from James Long Company. Segments were excluded if they contained excessive artifact due to eye blinks and muscle artifact, or if the EEG signal exceeded ±250 µV on any channel. The number of artifact-free trials averaged 16.4 (*SD* = 9.6) for the observe-hand group and 19.7 (*SD* = 6.6) for the observe-foot group.

Based on work showing that the infant mu rhythm oscillates with a peak frequency of 7–8 Hz towards the end of the first year and across the second year of life [27], [37]–[39], the mu frequency band was taken as 6–9 Hz. Previous work has shown power in this frequency range is reduced at central electrode sites during infants’ observation and execution of actions [23], [40]. Spectral power was estimated using Gaussian-tapered Morlet wavelets, and changes in power were computed as event-related spectral perturbation (ERSP) [41] during the observation of the experimenter’s reach to the object relative to a preceding baseline period.

The point of contact between the experimenter’s hand or foot and the object was taken as time zero (0 ms) in the analysis. Mean ERSP was computed from -800 ms to 0 ms, during which time the experimenter was reaching to the object. The baseline period extended from −1600 to −1100 ms, when the experimenter was sitting quietly prior to initiating her reach. Analyses focused on ERSP at electrode sites overlying the hand area (the left and right central electrodes; C3 and C4) and the foot area (the central midline electrode; Cz) of sensorimotor cortex. Preliminary analyses revealed no significant differences in ERSP between C3 and C4 and no differential patterns of results between these two electrodes. For the purposes of analyses, the ERSP values from C3 and C4 were therefore averaged to index mu rhythm activity over the hand areas.

## Results

**Figure 2:**
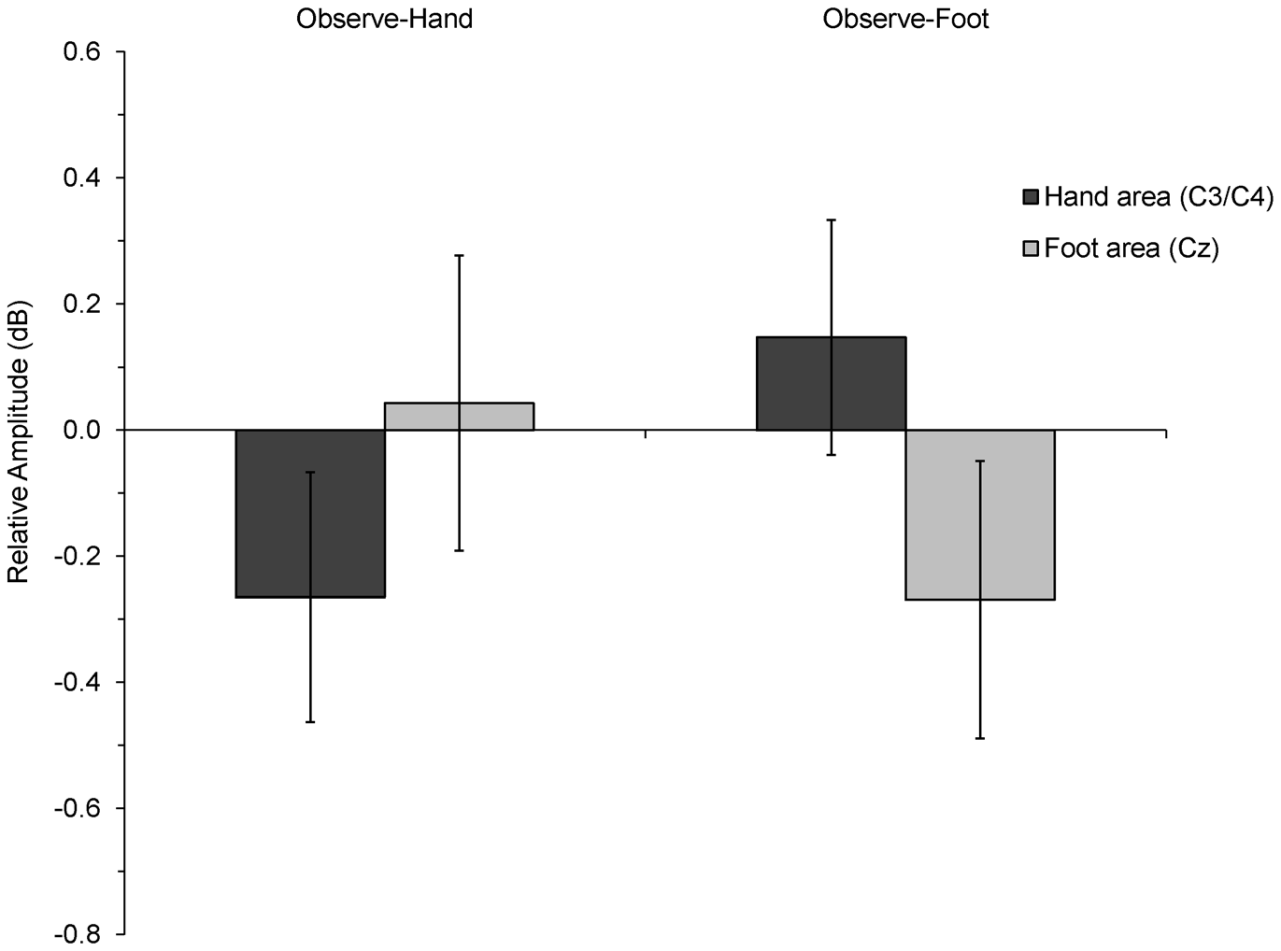
Mean relative amplitude (dB) in the mu band (6–9 Hz) during observation of the experimenter (prior to her touching the object). Negative values reflect a reduction in mu rhythm amplitude (desynchronization) and positive values reflect an increase in amplitude (synchronization) relative to a pre-stimulus baseline. Desynchronization patterns significantly varied as a function of experimental group. There was greater reduction in amplitude over the hand areas (C3/C4) for infants who observed hand actions; conversely, there was greater reduction in amplitude over the foot area (Cz) for infants observing foot actions. Error bars represent 1 SEM.

**Figure 3:**
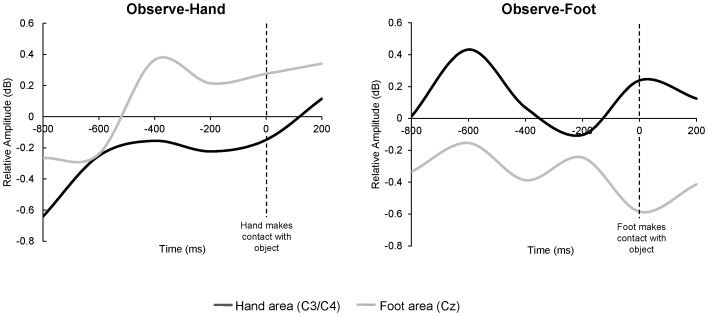
Mean relative amplitude (dB) in the mu band (6–9 Hz) at central sites as a function of time, during observation of the experimenter’s reach (prior to her touching the object). The zero point is the first video frame in which the experimenter touched the object. At central sites, which overlie sensorimotor cortex, patterns of activity varied as a function of whether infants observed the hand or the foot action. Desynchronization occurred over hand areas (C3/C4) for the Observe-Hand group; desynchronization occurred over foot area (Cz) for the Observe-Foot group.

Consistent with the prediction of neural somatotopy, a 2×2 ANOVA showed a significant interaction between electrode position (hand area vs. foot area) and group (Observe-Hand vs. Observe-Foot), *F* (1, 30) = 6.21, *p* = .018. There were no significant main effects of electrode position or group. As indicated by the data in Figure 2, the significant interaction is due to differential spatial patterns of mu rhythm activity between the two experimental groups. Specifically, the mu rhythm showed greater desynchronization over hand areas for the infants who observed hand actions, and greater desynchronization over the foot area for the infants who observed foot actions. Figure 3 plots the same underlying data as shown in Figure 2, but does so in a dynamic fashion showing the temporal unfolding of the mean ERSP over the course of the 800 ms reaching epoch (for plots of individual subjects, see Figure S1).

Although our primary predictions concerned the mu rhythm over the central scalp region, similar analyses were performed for the parietal region (electrodes P3/P4 and Pz), since some infant EEG studies involving observation of hand actions have reported findings at parietal sites as well as at central sites [24], [27]. No significant interaction between electrode position and group was noted over the parietal region (*F* (1, 30) = 0.31, *p*>.50), suggesting that the somatotopic response of the infant mu rhythm was specific to central sites.

## Discussion

We addressed a key question about the infant brain response to action observation: Is the pattern of desynchronization of the sensorimotor mu rhythm during observation of goal-directed acts sensitive to the specific means used by the actor? Adult work reveals a somatotopic organization both of hemodynamic responses to action observation using fMRI [7] and of EEG mu rhythm responses to action production and motor imagery [28], [31]. Work with infants has shown somatotopic EEG activity in response to direct tactile stimulation and their own motor activity [16], but no prior study has examined the possibility of infants’ somatotopic responses to the mere *observation of another’s action*.

Two randomly assigned groups of infants saw the same experimenter achieve the same goal, but one observed the experimenter use her hand and the other group observed her use her foot. The significant difference in spatial distribution of the sensorimotor mu rhythm response as a function of experimental group suggests a somatotopic organization of infant brain responses to action observation. Relative to activity over hand areas, desynchronization of the mu rhythm over the foot area of sensorimotor cortex was greater in the group of infants who observed foot actions than in the group who observed hand actions. Conversely, desynchronization over the hand area of sensorimotor cortex was greater for the infants who watched hand actions relative to those who observed foot actions.

Future work could extend these novel findings to test theories of action representation and developmental cognitive neuroscience. Critically, one could systematically vary infant self-experience with using one or another specific effector to accomplish a goal, and then assess changes in the somatotopic response to action observation based on this prior self-experience. One could also investigate novel effectors, such as using the elbow or head to push a button, although the reduced spatial distance between the corresponding areas of sensorimotor cortex may present challenges for conventional infant EEG work. In this respect, magnetoencephalography (MEG) technologies adapted for infants may be helpful since they could potentially provide more specific source localization data. Another avenue would be to investigate how neural somatotopy might be altered in children with neurodevelopmental disorders. One intriguing possibility is that children with autism spectrum disorder–who have deficits in imitation and interpersonal connection to other people [42]–[44] –may have a distortion or alteration in neural somatotopy, which could disrupt the development of self-other mapping at a basic level.

This study provides the first evidence that infants’ observation of an act produced by another person using a particular body part is associated with activation of the corresponding area of the infant’s own sensorimotor cortex. We believe this provides a neural correlate for what has been labeled as *organ identification* [15]. The finding of intercorporeal mapping provides connections between developmental social-cognitive neuroscience and two different literatures. First, it links to a body of work suggesting that the brain processes involved in observing other’s actions are closely linked to the processes involved in producing and monitoring one’s own actions [1], [3]. Second, it connects with behavioral studies of infant imitation showing that the representations of others’ acts preserves information about the specific body part used [14], [15]. The neural self-other mapping demonstrated here may support imitation and facilitate the rapid cultural learning that characterizes the human young.

## Supporting Information

Figure S1 **Data for one infant from each condition (Observe-Hand; Observe-Foot).** Although it is not standard practice in event-related EEG studies to display individual data in addition to group averages, this figure was provided upon the request of a reviewer. Relative amplitude (dB) is shown in the mu band (6–9 Hz) at central sites as a function of time during observation of the experimenter’s reach, prior to her touching the object. The zero point is the first video frame in which the experimenter touched the object.
(PDF)
