# Supplementary figures and images for: Infants’ Somatotopic Neural Responses to Seeing Human Actions: I’ve Got You under My Skin
Source: PLoS One. 2013 Oct 30;8(10):e77905. doi: 10.1371/journal.pone.0077905 (PMC3813772; doi:10.1371/journal.pone.0077905)

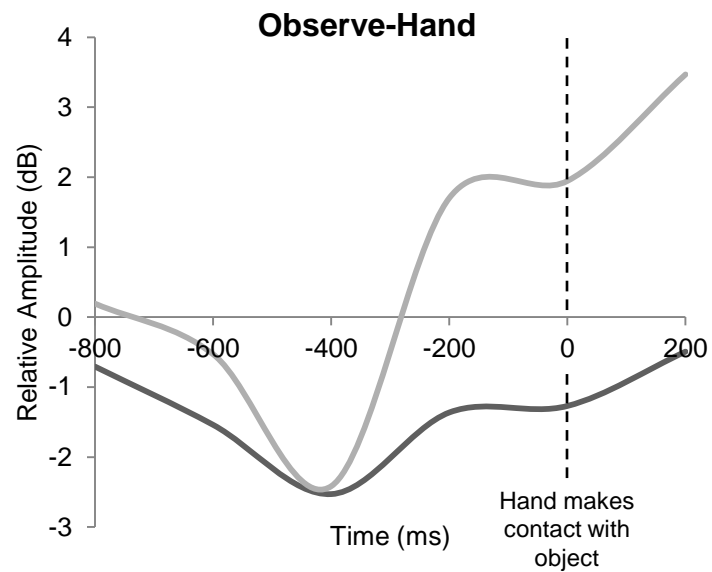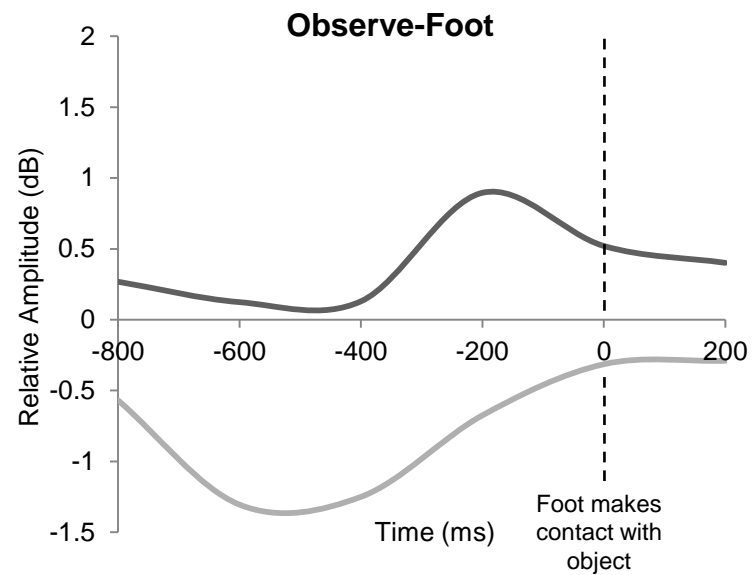

— Hand area (C3/C4) — Foot area (Cz)

Supplement: Figure S1 — Data for one infant from each condition (Observe-Hand; Observe-Foot). Although it is not standard practice in event-related EEG studies to display individual data in addition to group averages, this figure was provided upon the request of a reviewer. Relative amplitude (dB) is shown in the mu band (6–9 Hz) at central sites as a function of time during observation of the experimenter’s reach, prior to her touching the object. The zero point is the first video frame in which the experimenter touched the object. (PDF) [file pone.0077905.s001.pdf]
